# ISO/IEEE 11073 Treadmill Interoperability Framework and its Test Method: Design and Implementation

**DOI:** 10.2196/22000

**Published:** 2020-12-09

**Authors:** Zhi Yong Huang, Yujie Wang, Linling Wang

**Affiliations:** 1 School of Microelectronics and Communication Engineering Chongqing University Chongqing China; 2 Bioengineering College Chongqing University Chongqing China

**Keywords:** ISO/IEEE 11073-PHD, treadmill, standard frame model, test standard, sports health data

## Abstract

**Background:**

Regular physical activity is proven to help prevent and treat noncommunicable diseases such as heart disease, stroke, diabetes, and breast and colon cancer. The exercise data generated by health and fitness devices (eg, treadmill, exercise bike) are very important for health management service providers to develop personalized training programs. However, at present, there is little research on a unified interoperability framework in the health and fitness domain, and there are not many solutions; besides, the privatized treadmill data transmission scheme is not conducive to data integration and analysis.

**Objective:**

This article will expand the IEEE 11073-PHD standard protocol family, develop standards for health and fitness device (using treadmill as an example) based on the latest version of the 11073-20601 optimized exchange protocol, and design protocol standards compliance testing process and inspection software, which can automatically detect whether the instantiated object of the treadmill meets the standard.

**Methods:**

The study includes the following steps: (1) Map the data transmitted by the treadmill to the 11073-PHD objects; (2) Construct a programming language structure corresponding to the 11073-PHD application protocol data unit (APDU) to complete the coding and decoding part of the test software; and (3) Transmit the instantiated simulated treadmill data to the gateway test software through transmission control protocol for standard compliance testing.

**Results:**

According to the characteristics of the treadmill, a data exchange framework conforming to 11073-PHD is constructed, and a corresponding testing framework is developed; a treadmill agent simulation is implemented, and the interoperability test is performed. Through the designed testing process, the corresponding testing software was developed to complete the standard compliance testing of the treadmill.

**Conclusions:**

The extended research of IEEE 11073-PHD in the field of health and fitness provides a potential new idea for the data transmission framework of sports equipment such as treadmills, which may also provide some help for the development of sports health equipment interoperability standards.

## Introduction

In order to prevent noncommunicable diseases, the World Health Organization recommends that the world establish special actions to encourage and guide people to participate in more sports, and therefore released the global action plan on physical activity 2018-2030 [1]. To achieve this goal, people need to carry out scientific and effective exercise. Health management service providers usually develop special and personalized training programs for users, and collect user’s sports data through a series of sports and health equipment including treadmills, power cars, wearable devices, and so on. These data can be incorporated into the personal health record [2], and the treadmill data can be integrated into a personalized health management service system along with data from other sports and health equipment.

Therefore, we need to customize a data flow interoperability protocol suitable for treadmills, and the protocol should preferably have the same semantic syntax as the exchange protocol of other sports and health equipment under the framework of a large protocol family. In this way, we can make multiple sports and health equipment conform to the same data exchange format, which greatly reduces the integration difficulty and cost of personal sports data, and facilitates the comprehensive analysis of multiple sports parameters.

The ISO/IEEE 11073 personal health data standard is a set of standards that address the interoperability of personal health equipment (such as scales, blood pressure meters, blood glucose meters). The 11073-PHD protocol family provides a unified semantic grammar data exchange framework for medical device and personal health equipment.

11073-PHD defines an agent device role, which represents a device that provides sports health data, and transmits the obtained data to the master device; a manager device role, which receives sports health data from one or more slave devices by wireless or wired transmission. Thanks to the 11073 protocol, personal health equipment has a unified data transmission protocol at the application layer.

In the 11073-PHD protocol family, 11073-20601 [3] is an optimal exchange protocol, which establishes an abstract logical connection framework between the manager and the agent. This general modeling framework is composed of 3 core models: domain information model (DIM), service model, and communication model, which are respectively used for the semantic description of information and its interrelation and the abstract expression of access interface, definition of data access service, description of interaction behavior, and definition of session synchronization mechanism.

The existing 11073-PHD [4,5] framework helps to provide interoperability for health equipment; unfortunately, compared with the designing and development of equipment and applications in the area of disease management, less efforts had been made to address the demand in the field of health and fitness, which has led to the fact that it cannot effectively support the richer personalized training applications, nor can fully respond to the potential capabilities of various equipment in the sports ecology centered on treadmills. Besides, there are a lot of legacy treadmill devices in the existing sports equipment market [6]. It is a major trend to intelligently transform these inventory devices. If a set of widely applicable interoperable standards can be properly applied, it will greatly reduce the difficulty of equipment transformation and system integration, and provide a unified and standardized interface for system integrators and third-party application developers.

In summary, it is necessary to develop suitable interoperability standards for treadmills, but there is less research work in this field. The development of standards for treadmills based on the latest version of the 11073-20601 exchange protocol can fill the above gaps in a technically appropriate and cost-effective manner. At present, no related research or project implementation is available. Therefore, we plan to expand a set of data transmission protocols specifically suitable for treadmills based on the 11073-PHD protocol family, and design a set of data stream detection schemes that match the protocol.

## Methods

### Design of PHD-Based Treadmill Interoperability Framework

In the design of treadmill interoperability framework, the main work is to create a DIM. First, we determine the parameters that the treadmill may transmit, then map the data type to the 11073-20601 general framework, add the attribute type of the mapped object according to the parameter type, and finally, determine the corresponding attribute value. As for the service and communication models, there is not much difference from the definition in 11073-20601.

Personal information such as height, weight, and age, and also speed, heart rate [7], distance, and other data generated during exercise during the marked period are essential for the analysis of personal exercise conditions and the formulation of personalized exercise plans [8]. Through the design of the following treadmill objects, the user’s movement process can be mainly described, and each concept is briefly explained in the following sections.

#### Session

A session is similar to an envelope and contains all measures related to an activity scenario or an exercise scenario. Each exercise set defines the start date and time of the scenario and the activities and duration of the activities that the user participates in during the scenario.

#### Subsession

A subsession is similar to an envelope and contains all the metrics related to the session. Each sport item defines the start date, start time, and duration of the sport item, and also includes the activities that the user participates in during the duration of the sport item.

#### Age

The age is usually entered manually by the user. The agent can use the age for derivative calculations (eg, calculating the maximum recommended heart rate).

#### Weight

Weight is usually a setting manually entered by the user, although the device can measure it directly. The weight setting may be used by the device to derive calculations; for example, to calculate the energy consumed during jogging.

#### Height

The height is usually a setting manually entered by the user. The altitude setting may be used by the device to derive calculations, for example, to calculate BMI.

#### Distance

The distance defines the total distance covered since the start of the session or event. Distance can be specified as an actual distance concept, for example, meters or feet; it can also be specified as a more abstract concept, for example, the number of steps or the number of stairs climbed. In the latter case, the distance represented by MDC_DIM_STEP (11520) is equal to the step measurement.

#### Energy Consumption

Energy consumption refers to the amount of energy consumed since the start of a session or event.

#### Dynamic Heart Rate

Heart rate can be observed as the maximum value, minimum value, and average value of a movement or action, and can also be expressed as an instantaneous value. This rate is a key indicator of physical exertion. In particular, the observed maximum heart rate is an important observation value that may be used to calculate the user’s VO_2max_.

#### Slope

Slope indicates the steepness of the slope, which can be expressed as the minimum value, average value, or maximum value in the session or subsession, or it can be expressed as the instantaneous value. Positive values indicate uphill and negative values indicate downhill. Therefore, the minimum slope value represents the steepest downhill slope during a session or item.

#### Maximum Recommended Heart Rate

The maximum recommended heart rate [9] is usually manually entered by the user (or doctor) or calculated. The simplest estimation method is *h* = 220 – *a*, where *h* is the maximum recommended heart rate and *a* is the age. The maximum recommended heart rate can be used to provide background information for other values, such as the maximum heart rate value, minimum heart rate value, and average observed heart rate value that can be reached during an exercise set.

#### Program Identifier

This measured value identifies the exercise program used by a person during a session or item.

#### Session–Subsession–Start–Indicator

“Session–Subsession–start–indicator” is used to mark the start position of the continuously monitored session or subsession.

#### Speed

Speed adds additional contextual information to the ongoing movement and is used to capture the speed of the user through a distance. Speed can be reported as the minimum speed value, average speed value, or maximum speed value in a session or subsession, or as an instantaneous speed report.

#### Target Heart Rate Range

The target heart rate range [10] is the recommended heart rate for a certain session or subsession. Users can try to keep their heart rate within this range to achieve the preset exercise goal. When the user’s actual heart rate exceeds this range, the treadmill directly gives the user a prompt, or sends the corresponding event message to the manager. In a certain session or event, the user should try to keep his/her speed above the lower limit to reach the preset exercise goal.

#### Target Speed Lower Limit

The target speed lower limit is the minimum speed for a certain session or sport item. The user should try to keep his speed above the lower limit to reach the preset exercise goal. When the user’s actual speed exceeds this range, the treadmill directly gives the user a prompt, or sends the corresponding event message to the manager.

**Target Energy Consumption Lower Limit**

It indicates the minimum energy that should be consumed in a certain session or item. The user should try to consume more energy than the target value to reach the preset exercise goal. When the user’s energy consumption value exceeds this target value, the treadmill directly gives the user a prompt, or sends the corresponding event message to the manager.

#### User’s Exercise Standard and Health Status

According to the training goal set by the user in advance, the treadmill will send some key information related to the user’s exercise physiological state to the manager in the form of an event report, such as “exceeded the upper limit of the target heart rate range,” “reached target energy consumption lower limit” and other information.

#### Target Heart Rate Distribution Plan

It is set by several “heart rate range + duration” parameter groups. The user’s exercise goal is to control his/her heart rate within a specified heart rate range for a certain length of time. Each parameter group contains 3 elements in sequence: the lower limit of the target heart rate range, the upper limit of the target heart rate range, and the duration of the target heart rate range.

#### VO_2max_

The maximal rate of oxygen uptake (VO_2max_) is an important determinant of cardiorespiratory fitness and aerobic performance. VO_2max_ can be estimated indirectly based on the heart rate at rest (HR_rest_) and the heart rate at maximal exercise (HR_max_)[11].

VO_2max_ = (15.0 mL min^–1^ kg^–1^)·(HR_max_/HR_rest_)

### Construction of Treadmill DIM

#### Treadmill Object Instantiation

**Figure 1:**
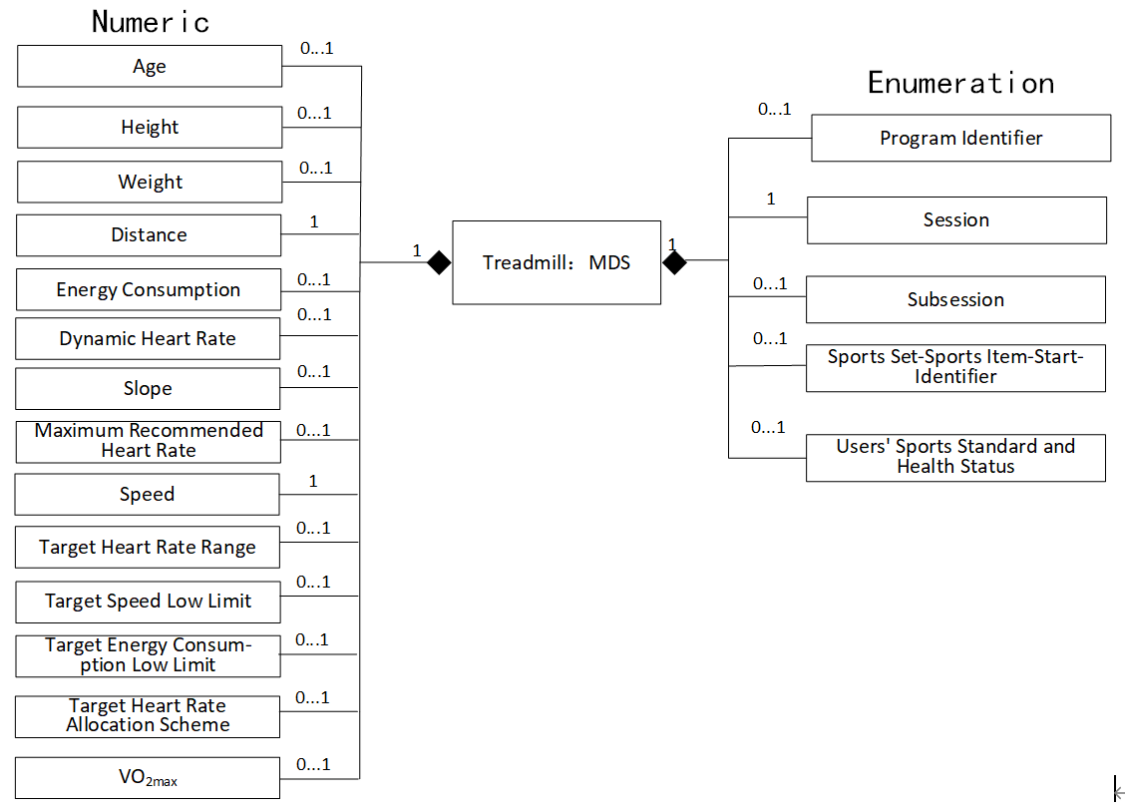
The object instance diagram of the treadmill DIM. DIM: domain information model; MDS: medical device system.

Complete the mapping of the parameters mentioned above to the numeric objects and enumerated objects defined by 11073-20601. The object example diagram is illustrated in Figure 1.

#### Design of the Main Attributes of the Object

For the object instance model related to device information characteristics, it is necessary to further design the attributes of the object, and to achieve the semantic representation of the device information characteristics carried by the object through the definition of attribute values [12]. Instanced objects can be divided into 2 categories: the first category is medical device system (MDS) objects representing context information, and the other category is metric-derived objects representing treadmill user data parameters.

##### MDS Object

The *Dev-Configuration-Id* attribute holds a locally unique 16-bit identifier that identifies the device configuration. The *System-Id* attribute is an IEEE EUI-64 address, consisting of a 24-bit organizationally unique identifier and a 40-bit manufacturer-defined ID [13]. The agent sends the Dev-Configuration-Id and System-Id to the manager in the “associated state,” so that the manager determines the configuration of the slave device during the association. If the manager has saved the configuration information related to *Dev-Configuration-Id* and *System-Id*, then it further identifies the *Dev-Configuration-Id* of the agent, and both agent and manager skip the “configuration state” and enter the “operating status.” However, if manager cannot recognize the *Dev-Configuration-Id* of the *System-Id*, then both agent and manager enter the “configuration state” [14].

The attribute value design of the MDS object is shown in Table 1.

**Table 1:** Object MDS’s attributes.

Attribute	The value of attribute
Handle	0
System-Model	{“Manufacturer”,“Model”}
System-Id	IEEE EUI-64 address
Dev-Configuration-Id	Extended configuration: 0x4000-0x7FFF
System-Type-Spec-List	Types and versions of device specifications: {MDC_DEV_SPEC_PROFILE_HF_CARDIO，3}; Device subtype and version: {MDC_DEV_SUB_SPEC_PROFILE_TREADMILL, 1}

##### Numeric Object

For the design of attribute values of numeric objects, the main aspects are the following:

- Handle: An unsigned, locally unique, 16-bit number, where each numeric object has a different nonzero handle value.
- Timestamp: All numeric object instances are associated with the session or subsession objects defined above. In the case of a session summary, only the session or subsession should have a timestamp attribute, whereas in the case of continuous monitoring of the session or subsession, the numerical object sampling instance not only reports the session summary attribute, but also each numerical object sampling instance brings its own timestamp attribute.
- Source-Handle-Reference: The session or subsession may contain associated numerical objects which represent observations that are generated throughout the session or subsession. Therefore, the Source-Handle-Reference attribute of a numeric object should identify whether the numeric object instance is associated with a session object or a subsession object. If the numeric object is an observation value at the session level, the Source-Handle-Reference attribute should be equal to the value of the handle of the session object. Similarly, if the numeric object is an observation value at the subsession level, the Source-Handle-Reference attribute should be equal to the value of the handle of the subsession object.
- BasicNuObsValue: In the numerical objects mentioned above, except for the target heart rate range and the target heart rate allocation plan, the basic numerical observations are all represented by the SFLOAT-Type type. Table 2 lists the design of Type, Metric-Spec-Small, and Unit-Code attribute values of other objects except the target heart rate range and target heart rate allocation scheme.

**Table 2:** Remaining attributes of numeric objects other than Target Heart Rate Range and Target Heart Rate Allocation Scheme.^a^

Object and type			Unit code
**Age**			
	MDC_HF_AGE (126)	MDC_DIM_YR (2368)	
**Height**			
	MDC_LEN_BODY_ACTUAL (57668)	MDC_DIM_M (1280)	
**Weight**			
	MDC_MASS_BODY_ACTUAL (57664)	MDC_DIM_KILO_G (1731)	
**Distance**			
	MDC_HF_DISTANCE (144)	MDC_DIM_M (1280) | MDC_DIM_CENTI_M (1278) | MDC_DIM_STEP (11520)	
**Energy Consumption**			
	MDC_HF_ENERGY (196)	MDC_DIM_CAL (8352) | MDC_DIM_JOULES (3968)	
**Dynamic Heart Rate**			
	MDC_HF_HR (180)	MDC_DIM_BEAT_PER_MIN (2720)	
**Speed**			
	MDC_HF_SPEED (168)	MDC_DIM_M_PER_SEC (2816) | MDC_DIM_CENTI_M_PER_MIN (6577) | MDC_DIM_STEP_PER_MIN (11616) | MDC_DIM_KILO_M_PER_HR (11939)	
**Target, Speed, and Low Threshold**			
	MDC_HF_SPEED_TARGET_LOW (2105)	MDC_DIM_M_PER_SEC (2816) | MDC_DIM_CENTI_M_PER_MIN (6577) | MDC_DIM_STEP_PER_MIN (11616)	
**Target Energy Consumption and Low Threshold**			
	MDC_HF_ENERGY_EXPENDED_TARGET_LOW (2109)	MDC_DIM_CAL (8352) | MDC_DIM_JOULES (3968)	
**VO_2max_**			
	MDC_HF_VO2_MAX (2112)	MDC_DIM_ML_PER_KG_MIN (4420)	
**Slope**			
	MDC_HF_INCLINE (176)	MDC_DIM_PERCENT (544) | MDC_DIM_ANG_DEG (736)	

^a^Metric-Spec-Small: mss-avail-intermittent | mss-avail-stored-data | mss-updt-aperiodic | mss-msmt-aperiodic | mss-acc-agent-initiated | mss-cat-setting.

The *Target heart rate range* object uses the *Compound-Basic-Nu-Observed-Value* attribute to transmit the lower and upper limit values of the Target heart rate range. The value of this attribute is only transmitted through a fixed format event report. When the treadmill sends a configuration report, it will report the *Attribute-Value-Map* attribute value of the target dynamic heart rate range. In the subsequent fixed format reports, the data content can be directly transferred according to that described in the *Attribute-Value-Map* without having to transfer the attribute Object Identifier [15] and the value length, which can reduce the length of the APDU to some extent. Here, the attribute sequence value of the *Attribute-Value-Map* is the attribute-id of the observation attribute, the timestamp attribute of the composite data, and the corresponding attribute value length. The Metric-Structure-Small attribute is used to identify each item of data in the observation list one by one. The order of the *Metric-Id-List* should correspond to the order of the observation items in the composite observation. Here, the first Object Identifier of the *Metric-Structure-Small* attribute value sequence is MDC_HF_HR_TARGET_LOW, and the second is MDC_HF_HR_TARGET_HIGH. For other attributes and their recommended attribute values, please refer to Table 3.

**Table 3:** Remaining attributes of the object Target Heart Rate Range.

Attribute	The value of attribute
Type	MDC_HF_HR_TARGET_RANGE (2100)
Metric-Spec-Small	mss-avail-intermittent | mss-avail-stored-data | mss-updt-aperiodic | mss-msmt-aperiodic | mss-acc-agent-initiated | mss-cat-setting
Metric-Id-List	First: MDC_HF_HR_TARGET_LOW (2101); Then: MDC_HF_HR_TARGET_HIGH (2102)
Metric-Structure-Small	ms-struct-compound(1)-multiple observations
Unit-Code	MDC_DIM_BEAT_PER_MIN (2720)
Attribute-Value-Map	MDC_ATTR_NU_CMPD_VAL_OBS_BASIC (2677) and MDC_ATTR_TIME_ABS (2439)
Compound-Basic-Nu-Observed-Value	It consists of 2 SFLOAT-Type dates: the first representing target heart rate low threshold and the other one representing high threshold.

The *Target heart rate allocation scheme* object is a data structure, which is set by several parameter groups of “heart rate range + duration + identifier.” The user’s exercise goal is to control his/her heart rate within a specified heart rate range for a certain length of time.

Each parameter group contains 3 elements in sequence: lower limit of the target heart rate range, upper limit of the target heart rate range, duration of the target heart rate range, and associated content identifier. The first 2 elements are provided by *Compound-Simple-Nu-Observed-Value*, the third element is provided by *Measure-Active-Period*, and the fourth element is provided by *Context-Key*. The value of this attribute is only transmitted via a fixed format event report. The following is an example of a heart rate distribution structure:

{

[70, 100, 180 seconds，“PLAN123”]

[100, 120, 240 seconds，“PLAN123”]

[120, 140, 120 seconds，“PLAN123”]

}

Table 4 illustrates the design of other attributes of the target heart rate allocation scheme.

**Table 4:** Remaining attributes of the object Target Heart Rate Aallocation Scheme.

Attribute	The value of attribute
Type	MDC_PART_PHD_HF|MDC_HF_HR_TARGET_ALLOC_PLAN
Metric-Spec-Small	mss-avail-intermittent | mss-avail-stored-data | mss-updt-aperiodic | mss-msmt-aperiodic | mss-acc-agent-initiated | mss-cat-setting
Metric-Id-List	First: MDC_HF_HR_TARGET_LOW; Then: MDC_HF_HR_TARGET_HIGH
Metric-Structure-Small	ms-struct-compound(1)-multiple observations
Unit-Code	MDC_DIM_BEAT_PER_MIN
Attribute-Value-Map	First: MDC_ATTR_NU_CMPD_VAL_OBS_SIMP; Second: MDC_ATTR_TIME_PD_MSMT_ACTIVE; Third: MDC_ATTR_CONTEXT_KEY (2680)
Compound-Simple-Nu-Observed-Value	Refer to the text description above.
Measure-Active-Period	The length of the period that each target range in the Target Heart Rate Aallocation Scheme lasts.
Context-Key	The value of this attribute is used to encode and identify different *Target Heart Rate Aallocation* to indicate the difference. Each target range that belongs to the same set of target heart rate allocation schemes uses the same identifier.

##### Enumeration Object

Table 5 illustrates the attribute value design of enumerated objects, and Table 6 lists the observed values of enumerated objects.

**Table 5:** Attributes of enumeration objects.

Object and attribute		The value of attribute
**Program Identifier, Session, Subsession, Session-Subsession-Strat-identifier, Users’ Sports Standard and Health Status**		
	Handle	An unsigned locally unique 16-bit number.
	Type	MDC_HF_PROGRAM_ID (108); MDC_HF_SESSION (123); MDC_HF_SUBSESSION (124); MDC_HF_STRT (125); MDC_HF_USER_FITNESS_HEALTH_STAT (126)
	Metric-Spec-Small	mss-avail-intermittent | mss-avail-stored-data | mss-updt-aperiodic | mss-msmt-aperiodic | mss-acc-agent-initiated.
	Absolute-Time-Stamp	See the description of the timestamp attribute of the previous numeric object.
	Measure-Active-Period	A FLOAT-Type that defines the length of the observation period (in seconds).
	Enum-Observed-Value-Simple-Oid (only Object Program Identifier owns)	The value is a free string type and is not restricted by any nomenclature.
	Enum-Observed-Value-Simple-Oid (This attribute is owned by all objects except Program Identifier.)	Refer to Table 6.
	Source-Handle-Reference	Refer to the footnote.^a^

^a^Source-Handle-Reference: For objects such as Program Identifier, Session-Subsession-Strat-identifier, Users’ Sports Standard and Health Status, their Source-Handle-Reference attribute value is the handle of Session or Subsession related to themselves; Subsession’s Source-Handle-Reference attribute value is the handle of the Session associated with itself; Session does not have this attribute.

**Table 6:** Observations of enumeration object.

Object and identifier		Semantic
**Session, Subsession, Session-Subsession-Strat-identifier**		
	MDC_HF_ACT_REST (1001)	Rest
	MDC_HF_ACT_UNKNOWN (1007)	Unknown
	MDC_HF_ACT_MULTIPLE (1008)	Mix of multiple types of sports
	MDC_HF_ACT_RUN (1011)	Jogging
	MDC_HF_ACT_WALK (1017)	Walk
	MDC_HF_ACT_WATER_WALK (1028)	Walking under water
**Users’ Sports Standard and Health Status**		
	MDC_HF_STAT_LT_HR_TARGET_LOW (2200)	The user’s heart rate is below the lower limit of the target heart rate range.
	MDC_HF_STAT_HT_HR_TARGET_HIGH (2203)	The user’s heart rate is above the upper limit of the target heart rate range.
	MDC_HF_STAT_HT_SPEED_TARGET_LOW (2207)	The user’s speed is higher than the target speed lower limit.
	MDC_HF_STAT_HT_ENERGY_EXPENDED_TARGET_LOW (2217)	The user’s energy consumption has exceeded the target energy consumption lower limit.

### Standard Compliance Testing Process

Because the above data transmission framework is derived from the 11073-20601 optimization exchange protocol, it is necessary to determine whether the data stream sent by the instantiated object that implements this standard meets the 20601 standard [16]. If the instantiated object of the treadmill interoperability framework passes the test, it indicates that the content it sends can have the same semantic grammar as the information sent by other devices that have met the 11073-PHD protocol family [17]. The testing content of this article will focus on the 3 models [18,19] of 11073-PHD, namely, (1) PHD DIM, (2) PHD service model, and (3) PHD communication model.

The test of DIM is mainly based on the events of MDS.

- *MDS-Configuration-Event*: If the manager cannot learn the current agent configuration information from the associated historical records, the agent sends the event to the manager during the startup of the “configuration” state. This event provides static information about the measurement functions supported by the agent.
- *MDS-Dynamic-Data-Update-Var*: This event provides dynamic data (usually measurement data) from the agent for the objects supported by the agent, and reports the object’s data in the format of a common attribute list variable.
- *MDS-Dynamic-Data-Update-Fixed*: Use the fixed format defined by the Attribute-Value-Map attribute of the measured object or MDS object to report data. The specific test items are shown in Multimedia Appendix 1 (see the “DIM test” section).

The service model provides the basic function of data access sent between the agent and the manager, and is used to exchange data derived from the DIM. The inspection items mainly include the command to obtain MDS device information (GET) and data report (Event Report). The specific test items are shown in Multimedia Appendix 1 (see the “SER test” section) [20].

The connection state machine defines a series of states and substates experienced between the agent and manager, including states related to connection, association, and operation. The communication model also defines the entry, exit, and error conditions of various states during the various running processes of measurement data transmission, which should be detected. The specific test items are illustrated in Multimedia Appendix 1 (see the “COM test” section).

### Test Software Framework Design

#### Module Design

The test software is mainly divided into 5 modules: Abstract Syntax Notation One (ASN.1) [21] module, encoding module, decoding module, communication module, and test module.

- The ASN.1 module, which defines all data types and data structures of C struct, reuses the ASN.1 code block in the Continua Enabling Software Library (CESL) [22] open source software package provided by Continua in the test software we designed.
- The encoding module generates an APDU binary data stream according to the instantiated APDU object and the Medical Device Encoding Rules used in 11073-20601.
- The decoding module, which refers to the ASN.1 module, converts the binary data stream of the data buffer into an instantiated APDU structure.
- The communication module adopts the abstract factory pattern, calls different subclass factories to produce and initialize instantiated objects of different underlying connection methods, and establishes data connections under the application layer.
- The test module will carry out the testing procedures according to the instantiated object returned by the decoding module, and generate a test result report.

#### Data Receiving and Testing Process

The data stream sent by the treadmill is transmitted to the application layer listening port of the test software via transmission control protocol (TCP)/USB/Bluetooth/Zigbee or other methods, and then the instantiated object produced by the communication module abstract factory [23] calls the message receiving function to store the binary stream into the data buffer. The decoding module refers to the APDU structure of the ASN.1 module and decodes the binary stream, and then generates the C++ instantiated object of the APDU. The test module calls application programming interface functions according to the designed test items, extracts the data related to the test items from the APDU instantiated objects for testing, and finally generates a test report.

#### Data Transmission Process

**Figure 2:**
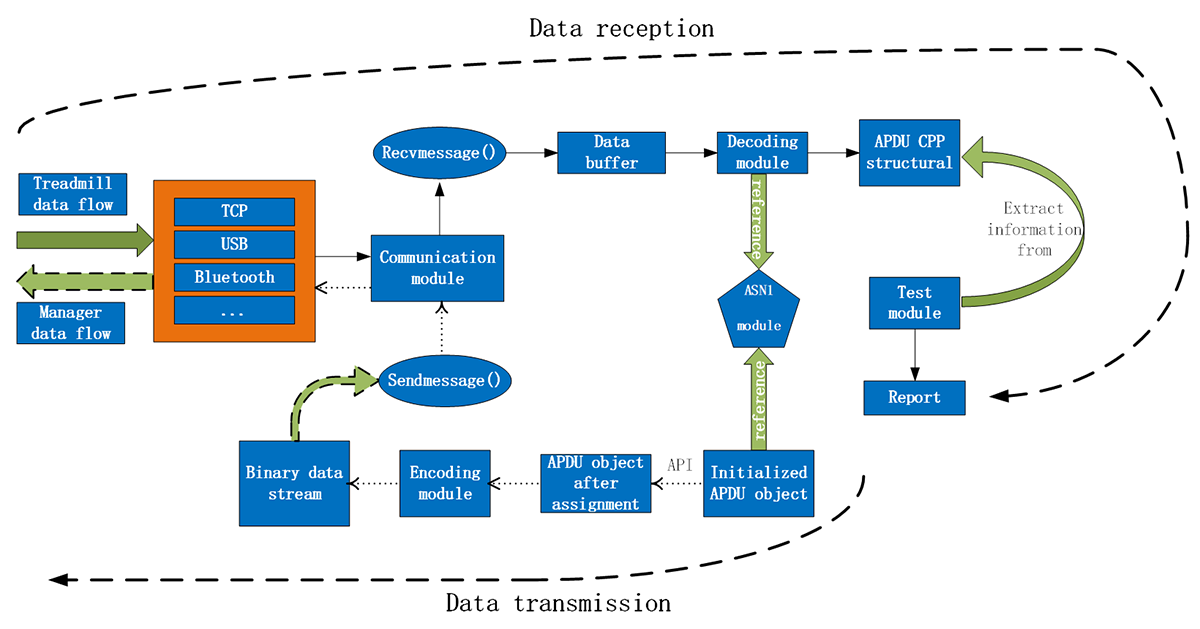
The process of receiving and sending data streams in the test software. APDU: application protocol data unit; TCP: transmission control protocol.

According to the APDU to be sent, refer to the ASN.1 module to establish the initialization APDU object, and then call the application programming interface function to assign the initialization object. The encoding module uses the Medical Device Encoding Rules to encode the assigned APDU object and generate a binary data stream. The communication module calls the message sending function to send the data to the simulated treadmill. The entire workflow of the test software is shown in Figure 2.

## Results

### Implementation of Treadmill Interoperability Framework

**Figure 3:**
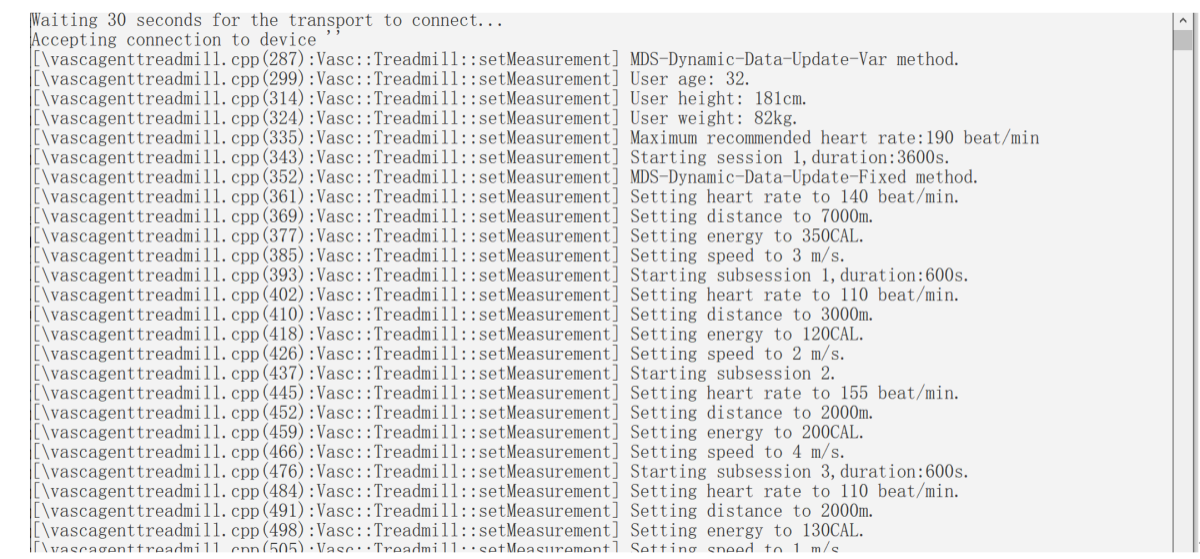
Information sent by simulated treadmill.

To verify the feasibility of the above standards, we built a simulated treadmill device based on the CESL open source software package. The treadmill device transmits the age, height, weight, maximum recommended heart rate, and other information once using the MDS-Dynamic-Data-Update-Var method (variable format data report); the MDS-Dynamic-Data-Update-Fixed (fixed format data report) method is used to transfer the Session and Subsession, dynamic heart rate, speed, energy consumption, and other information multiple times. The fixed format data report eliminates the description information such as data length and attribute ID. This is because the treadmill includes its own data format context in the configuration report and sends it to the test software before reaching the operating state. For fixed data sent periodically, fixed format data reports can save some byte streams. Figure 3 shows the data sent to the test software by the simulated treadmill acting as an agent.

### Testing Software

**Figure 4:**
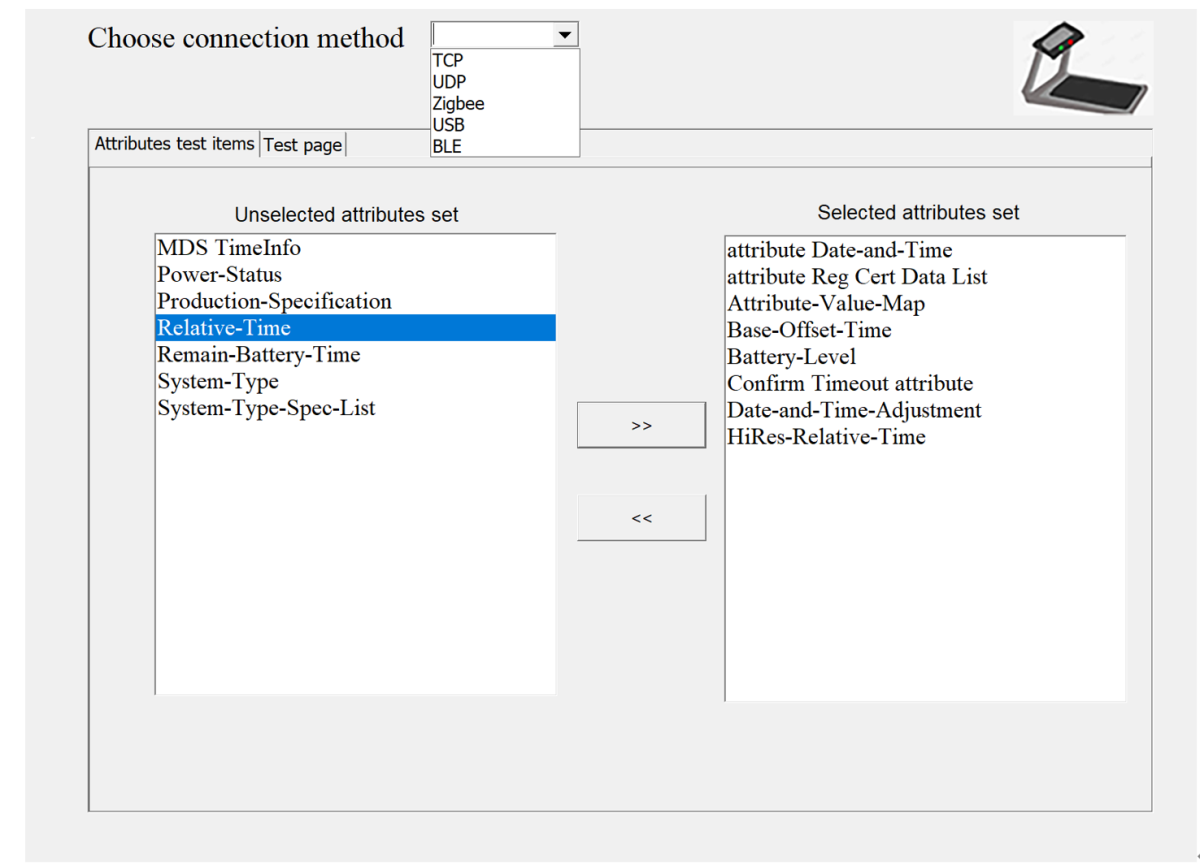
Test software start interface.

Here, the test software also plays the role of a manager, receiving the data stream sent by the treadmill to the binding port through the socket communication method of TCP, completing the test work according to the process, and then generating the final test result set report. The test software provides TCP, user datagram protocol, Zigbee, and other low-level interface connection methods, and provides optional MDS test attributes in the initial interface. Figure 4 shows the initial interface of the test software, selecting the connection method and test attributes.

**Figure 5:**
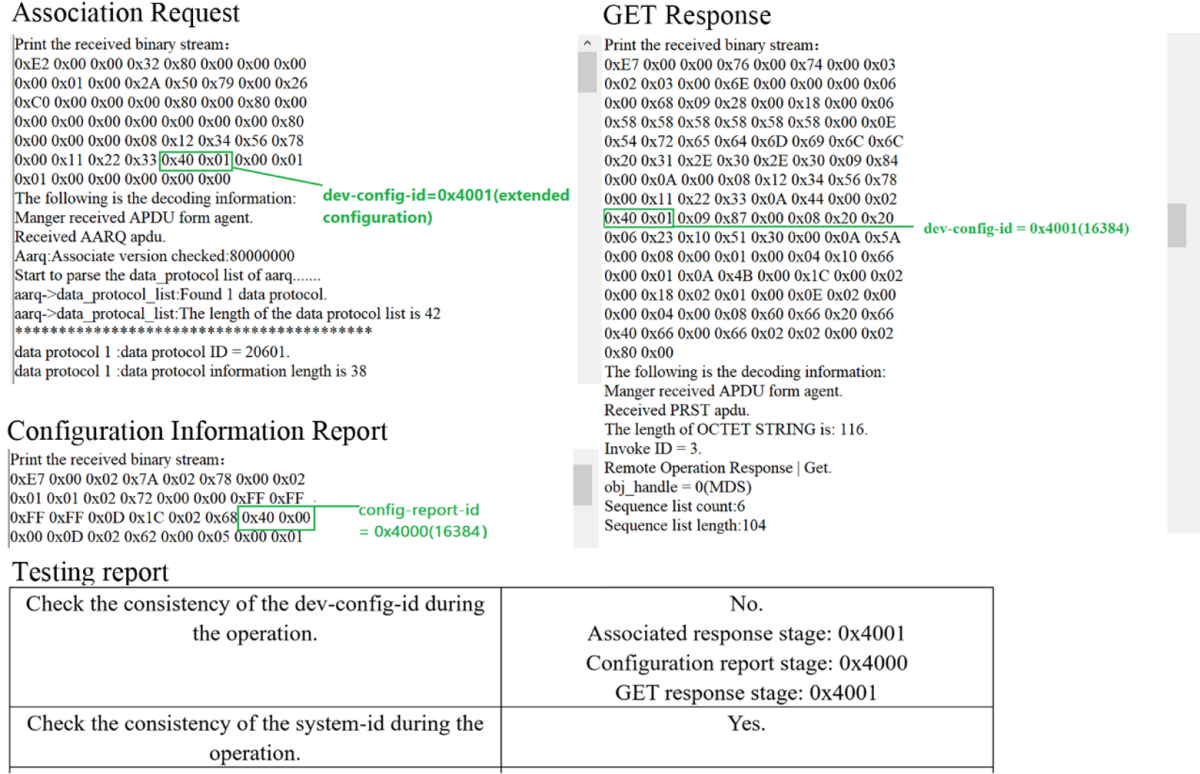
The value of dev-config-id in different APDUs and its consistency test results. APDU: application protocol data unit.

Figure 5 illustrates the test result of device configuration ID. During the association between an agent and a manager, the value of dev-config-id in the “Association Request” message indicates the configuration that the agent wants to use. In the subsequent “Configuration Information Report” and “GET Response” APDU, dev-config-id value should be consistent. In the APDU sent by the simulated treadmill, we deliberately set the value of the dev-config-id in “Association Request” and “GET Response” to 0x4001, and set the value of the dev-config-id in the “Configuration Information Report” to 0x4000. As can be seen in the test report generated by the test software, the consistency check item of dev-config-id has not passed, and it is given its value in the respective APDU.

**Figure 6:**
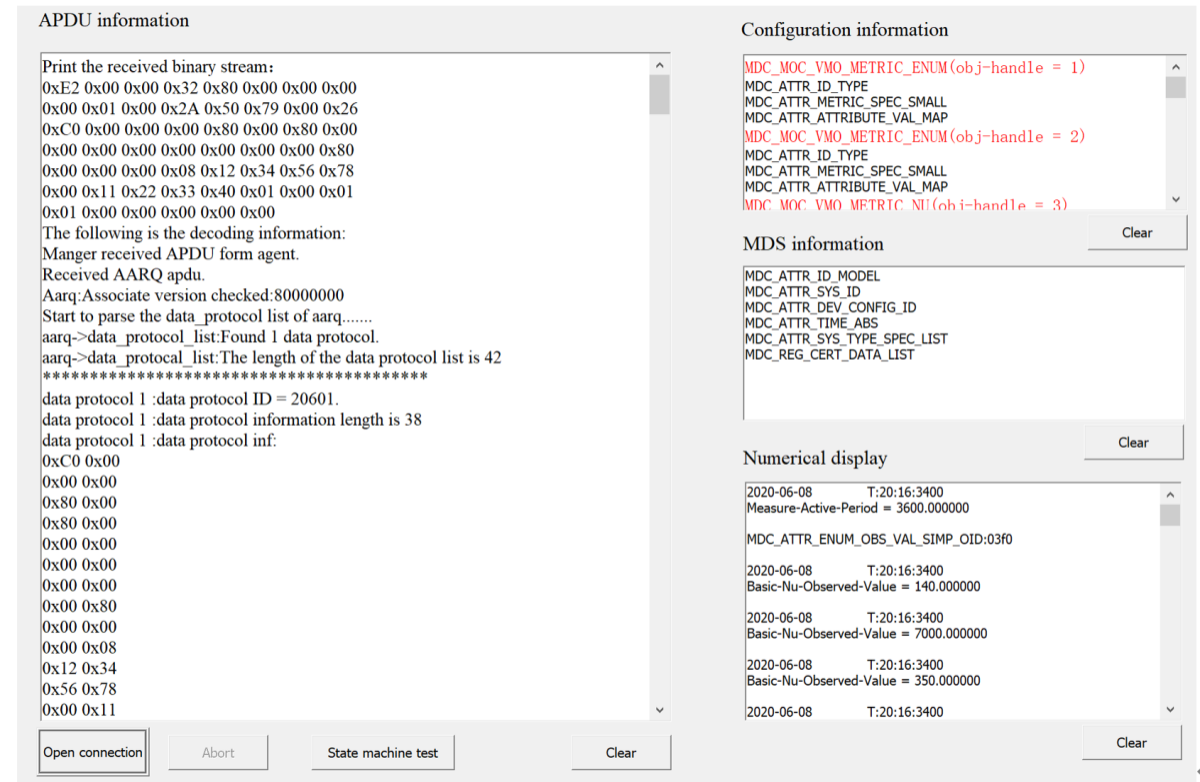
Data transmission between test interface and treadmill.

**Figure 7:**
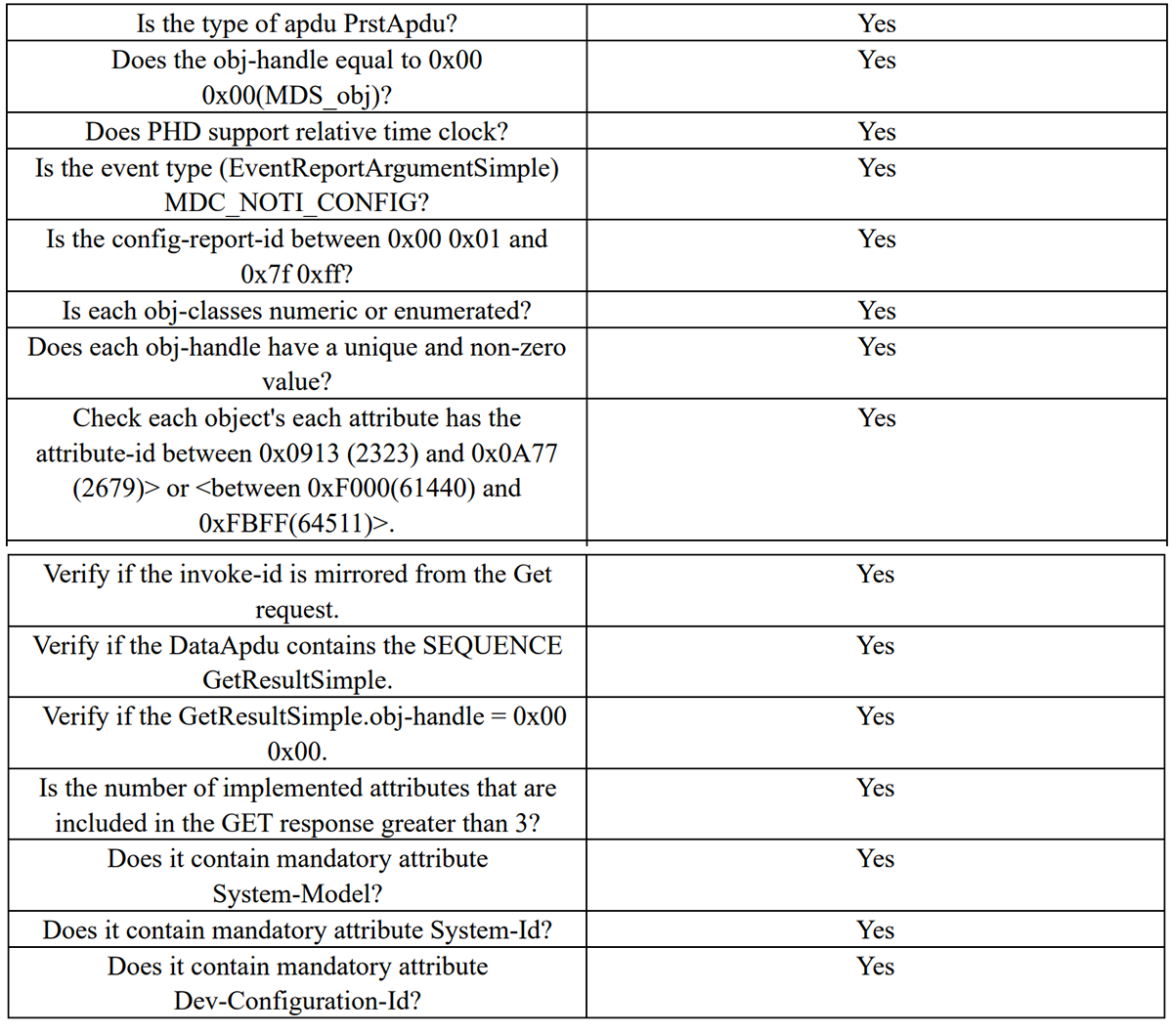
Part of the test results.

Figure 6 shows the ongoing communication process between the test software and the treadmill. In the large box on the left side of the interface, we can see the binary data stream and partial decoding information of each APDU in real time; the first small box on the right side of the interface is the objects and attributes contained in the configuration report sent by the treadmill; the second small box is the attribute information of the MDS object; the third small box presents the observation value sent by the treadmill and the corresponding timestamp in real time. After the routine test is completed, the state machine test button is clicked to perform the state machine test. After all the test items are tested, a test report will be generated, and the results will be displayed in a list. Figure 7 demonstrates a small part of the results of the final test report.

## Discussion

In this article, we propose a treadmill data interoperability protocol based on 11073-PHD, and design a set of standard compliance testing methods that match it. Using the testing software, we tested the data stream sent by the simulated treadmill equipment and generated a corresponding test result report.

In previous work, most manufacturers of sports and health equipment such as treadmills have their own set of data transmission standards, which is very unfavorable for data integration analysis and processing between different manufacturers and different sports and health equipment. In our work, through tailoring and customizing the existing 11073-PHD, we designed a set of protocol standards suitable for the transmission of treadmill data. This not only provides a possibility to unify the data transmission standards of treadmill equipment among various manufacturers, but more importantly, it also provides an idea for unifying the application layer data format of other sports and health equipment. Sports health equipment is designed based on the 11073-PHD-based customized design, so that they have the same semantic syntax, making it possible for a gateway device to integrate multiple sports health data.

We have investigated 4 popular treadmill private protocols used in the market to transmit key data (Table 7), and compared all their functions with the standard protocols we developed. While Hlink’s running posture detection data have no corresponding functional objects, the key data-bearing function objects established by our interoperability framework can cover all the main data of the 4 devices. A unified semantic syntax can help expand and upgrade service capabilities, which may greatly facilitate remote data capture, thereby enhancing the remote interaction between service providers and users.

**Table 7:** Comparison of proprietary protocols and standards.

Private standard and key data		Standard object
**SOLE**		
	Pulse (beats/minute)	Dynamic heart rate (beats/minute)
	Distance (km)	Distance (km)
	Calories	Energy consumption (kcal)
	User profile	Age (years), weight (kg), height (cm), user’s exercise standard and health status
	Program name	Program identifier
	Speed (km/h)	Speed (km/h)
	Slope (degree)	Slope (degree)
**Hlink (HUAWEI)**		
	Calories	Energy consumption (kcal)
	Heart rate (beats/minute)	Dynamic heart rate (beats/minute)
	Distance	Distance (km)
	Speed	Speed (km/h)
	Steps	Distance (steps)
	Program	Program identifier
	Running posture	—^a^
**Keep**		
	Maximum heart rate (beats/min)	Maximum recommended heart rate (beats/min)
	Sports set	Session
	Calories	Energy consumption (kcal)
	Step frequency	Speed (steps/min)
	Speed	Speed (km/h)
	Distance	Distance (km)
**IOT (XIAOMI)**		
	Speed	Speed (km/h)
	Distance	Distance (km)
	Steps	Distance (steps)
	Calories	Energy consumption (kcal)
	Mode	Program identifier
	Slope (%)	Slope (%)

^a^—: not available.

However, this work plan only supports some common data information functions of treadmills in the usual sense. Some plans, such as Hlink’s running posture detection, are not completely covered. This requires a more comprehensive arrangement and improvement in the next step. In addition, the treadmill we define is just acting as an agent. However, if you add some additional equipment that can be connected to a treadmill, such as a sports watch, the treadmill plays a dual role. When the treadmill is responsible for receiving data from the sports watch, it acts as a master device; at the same time, the treadmill transmits all its data to the gateway device. At this time, it acts as an agent device. The above situation covers only a small number of applications in the treadmill market, and our standard is only applicable for treadmills with common features at this stage. Finally, there is a lack of information expression regarding the working state of the treadmill itself (the working state of the electronic control board and the working state of the sensing components). Further information describing whether the speed and slope adjustment unit is working properly can be added.

## Appendix

Multimedia Appendix 1 Testing method.

## Abbreviations

APDU: application protocol data unit
ASN.1: Abstract Syntax Notation One
CESL: Continua Enabling Software Library
DIM: domain information model
HR_max_: heart rate at maximal exercise
HR_rest_: heart rate at rest
MDS: medical device system
TCP: transmission control protocol
