# Supplementary material for: ISO/IEEE 11073 Treadmill Interoperability Framework and its Test Method: Design and Implementation
Source: JMIR Med Inform. 2020 Dec 9;8(12):e22000. doi: 10.2196/22000 (PMC7758169; doi:10.2196/22000)
Supplement: Multimedia Appendix 1 [file medinform_v8i12e22000_app1.docx]

**Testing method**

#### DIM test

(1) *Dev-Configuration-Id and System-Id semantic feature.*

Check that: The Dev-Configuration-Id is consistent between the Configuring state and the Operating state. [AND] The PHD uses a “Remote Operation Invoke | Confirmed Event Report” data message with an event-type of MDC_NOTI_CONFIG to send its configuration to the PHG [23].

(2) *Static Numeric/ Enumerated attributes derived from Metrics class and Dynamic Numeric/ Enumerated attributes.*

Check that: Numeric/ Enumerated class is derived from the Metric base class. It inherits all mandatory attributes and conditional attributes as required by their conditions and it may import optional attributes. [AND] The nomenclature code to identify the Numeric class is MDC_MOC_VMO_METRIC_NU/MDC_MOC_VMO_METRIC_ENUM.

(3) *Numeric attributes: Metric-Id-List Semantic.*

Check that: The order of the Metric-Id-List shall correspond to the order of the elements in the compound observed value.

(4) *MDS objects methods and events. PHD data transmission.*

Check that: If the PHD uses Variable/ Fixed Format Event Reporting and reports on a single treadmill user, then it uses the MDS-Dynamic-Data-Update-Var/ MDS-Dynamic-Data-Update-Fixed Event to report dynamic data and the type of the Data APDU is ScanReportInfoVar/ ScanReportInfoFixed. [OR] If the PHD uses Variable/ Fixed Format Event Reporting and reports on multiple treadmill users, then it uses the MDS-Dynamic-Data-Update-MP-Var/ MDS-Dynamic-Data-Update-MP-Fixed Event to report dynamic data and the type of the Data APDU is ScanReportInfoMPVar/ ScanReportInfoMPFixed.

(5) *MDS object events. PHD configuration event.*

Check that: MDS object sends the MDS-Configuration-Event with an Event-Info parameter of type ConfigReport. Only confirmed mode. [AND] Each object shall have a unique identifier assigned by the PHD.

(6) *MDS Object: Mandatory, Conditional and Optional Attributes.*

Check that: The Personal Health Device (PHD) supports a Get command that requests all attributes [AND] The PHD reports its MDS object attributes to the Personal Health Gateway (PHG) using a Data message with the “Remote Operation Response | Get” response. [AND] MDS object contains all mandatory attributes, conditional attributes as required by their conditions and it may contain optional attributes [AND] MDS object attributes are static /dynamic or observational. [AND] The handle is entered in the obj-handle field and it is not included in the attribute ID list of the request or in the attribute list of the response. [AND] The MDS object is not considered part of the configuration.

#### SER test

(1) *Object Access Services: No-Such-Action Error*.

Check that: If a request for a confirmed action is received by a PHD that does not support the action, the PHD replies with an error (roer) value of no-such-action.

(2) *Configuration event report: dev-configuration-id is locally unique.*

Check that: If PHD has multiple device configurations, the assigned Dev-Configuration-Id values are locally unique [AND] The PHD transfers its configuration the PHG using a configuration event report [AND] The PHD consistently uses the Dev-Configuration-Id for subsequent associations. [AND] If the PHD receives an unsupported configuration message, the PHD sends a further configuration. This process is repeated until the PHD has attempted all configurations. When it sends an Association Release message with a reason code of no-more-configurations to indicate that it is unable to operate with the PHG the PHD moves to Disassociating state.

[AND] The same Dev-Configuration-Id shall not be used by an PHD for subsequent associations to identify a different device configuration. [AND] An PHD should use the same value for Dev-Configuration-Id in future Association Requests with the PHG to denote the same configuration of the device.

(3) *PHD transmits data in a fixed format Event Report*.

Check that: If PHD transmits data in fixed format, then it reports the object handle and the attribute values are in the same order and size as specified in the Attribute-Value-Map [AND] These Attribute-Value-Map attribute shall be defined and transmitted to the PHG before fixed format event report transfer commences [AND] The order of these elements is defined by the order in which the attribute identifiers are listed in the Attribute-Value-Map. The PHD controls the order and communicates it to the PHG via the Attribute-Value-Map attribute.

(4) *PHD transmits data in variable format Event Report*.

Check that: If PHD transmits data in variable format, then the event report fits to specified format.

#### COM test

(1) *Association request format*.

Check that: If a PHD sets the data-proto-id to data-proto-id-20601, then it adheres to the abstract syntax definitions specified for data types and message exchange. [AND] The data-proto-info field is filled in with a PhdAssociationInformation structure which defines the following information: -The version of the data exchange protocol. - The specific Data Apdu encoding rule(s) supported by the PHD. The PHD sets one or more of the encoding-rules bits. - The PHD always supports Medical Device Encoding Rule (MDER). That is, the MDER bit of encoding-rules is set by the PHD. - The PHD may offer other encoding rules, besides MDER, to the PHG by setting other bits in the encoding-rules. - The version of the nomenclature used a field indicating all functional units and optional features supported by the PHD. - The system type (PHD in this case). - A unique System-Id of the PHD. The PHD uses EUI-64 to identify itself. - A dev-config-id, which identifies the current configuration of the PHD. - A data-req-mode-capab, which defines the data request modes supported by the PHD. - An option-list that contains a list of additional attributes the PHD wishes to communicate [14]. [AND] The PHD shall place at most one data-proto element containing the field data-proto-id set to data-proto-id-20601 in the data-proto-list.

(2) *Agent State machine. Association timeout*.

Check that: If timeout and maximum retry limit are not reached while in the Associating state, then the PHD continues transmitting aarq. [AND] In the case of timeout, the PHD attempts to associate up to the maximum retry count is reached or association is successful. [AND] If timeout and maximum retry limit are reached when sending aarq, then the PHD transmits an abort message abrt(Abort-reason response-timeout) and moves to the Unassociated state.

(3) *Agent State machine. Connected Associating*.

Check that: If aarq is received while in Associating state, then the PHD transmits an aare(rejected permanent) and moves to the Unassociated state. If rlrq is received, then the PHD transmits an abort message abrt(Abort-reason undefined) and moves to the Unassociated state. If rlre is received, then the PHD transmits an abrt(Abort-reason undefined) and moves to the Unassociated state. If prst (any APDU not covered in 3.* (corrupt, unknown, unexpected, etc.) is received, then the PHD transmits an abort message abrt (Abort-reason undefined) and moves to the Unassociated state.See Table 8 for a detailed summary.

| **Table 8 . Agent State machine- Connected Associating** | | |
| --- | --- | --- |
| **Initial condition:** **The PHD under test is in the connected Associating state.** | | |
| **Apdu received by agent** | **Agent's next state** | **Agent's response apdu** |
| Association-request | Unassociated state | Association-response(rejected permanent) |
| Association-response |  | Abort (Abort-reason undefined) |
| Release-request |  | Abort (Abort-reason undefined) |
| Release-response |  | Abort (Abort-reason undefined) |
| Presentation |  | Abort (Abort-reason undefined) |

(4) *Agent State machine. Connected Associated*.

Check that: If aarq is received while in the Waiting Approval state, then the PHD transmits an abrt (reason undefined) and moves to Unassociated state. If aare is received, then the PHD transmits an abrt (reason undefined) and moves to the Unassociated state. If rlrq is received, then the PHD transmits a rlre and moves to the Unassociated state. If rlre is received, then the PHD transmits an abrt (reason undefined) and moves to the Unassociated state.

If roiv-* but not (roiv-cmip-get, handle=0) is received, then the PHD transmits a roer (no-such-object-instance) and remains in Waiting Approval state. If rors-*, roer-*, or rorj-*,but not rors-cmip-confirmed-event-report is received while PHD is in "Waiting Approval" state, an PHD shall transmit an abrt(reason undefined) and move to the Unassociated state. If rors-*, roer-*, or rorj-*,but not rors-cmip-confirmed-event-report is received, an PHD shall transmit an abrt(reason undefined) and move to the Unassociated state. Table 9 summarizes these situations.

| **Table 9 . Agent State machine- Connected Associated** | | |
| --- | --- | --- |
| **Initial condition:** The PHD under test is in the connected associated configuring Waiting Approval state. | | |
| **Apdu received by agent** | **Agent's next state** | **Agent's response apdu** |
| Association-request | Unassociated state | Abort (Abort-reason undefined) |
| Association-response |  | Abort (Abort-reason undefined) |
| Release-request |  | Release-response |
| Release-response |  | Abort (Abort-reason undefined) |
| Remote Operation Invoke \| Get | Operating state/ Waiting Approval state. | See the analysis below the table for details. |
| roiv-* but not (roiv-cmip-get, handle=0) | Waiting Approval state. | Remote-Operation-error(no-such-object-instance) |

If handle=0 is received while in Waiting Approval state, then the PHD transmits an rors-cmip-get with the MDS attributes then move to operating state or roer not-allowed-by-object if request is not for all attributes and PHD does not support the request and PHD remains in Waiting Approval state. [AND] If the PHG requests specific MDS object attributes, as indicated by the elements in attribute-id-list, and if this capability is not implemented, then the PHD shall respond with an error (roer) message with an error-value of not-allowed-by-object and PHD remains in Waiting Approval state.

(5) *Agent State machine. Leaving the Operating State*.

Check that: If aarq is received while in the Operating state, then the PHD transmits an abrt(Abort-reason undefined) and moves to Unassociated state; If aare is received, then the PHD transmits an abrt(Abort-reason undefined) and moves to the Unassociated state. If rlrq is received , then the PHD transmits an rlre (normal) and moves to the Unassociated state. If rlre is received ,then the PHD transmits an abrt(Abort-reason undefined) and moves to the Unassociated state. Refer to Table 10 for details.

| **Table 10 . Agent State machine-Leaving the Operating State** | | |
| --- | --- | --- |
| **Initial condition: The simulated PHG and PHD under test are in the Operating state.** | | |
| **Apdu received by agent** | **Agent's next state** | **Agent's response apdu** |
| Association-request | Unassociated state | Abort (Abort-reason undefined) |
| Association-response |  | Abort (Abort-reason undefined) |
| Release-request |  | Release-response（normal） |
| Release-response |  | Abort (Abort-reason undefined) |

(6) *Disassociating procedure*.

The Association Release Request contains a ReleaseRequestReason with reason = normal or configuration-changed or nomore-configurations to indicate the reason for releasing the association. When the PHD sends an Association Release message and waits for an Association Release Response message for a TOrelease period without receives it, then the PHD sends an Association Abort message and moves to Unassociated state.
